# Supplementary material for: A renal YY1-KIM1-DR5 axis regulates the progression of acute kidney injury
Source: Nat Commun. 2023 Jul 17;14:4261. doi: 10.1038/s41467-023-40036-z (PMC10352345; doi:10.1038/s41467-023-40036-z)
Supplement: Supplementary file 1 — supplementary information [file 41467_2023_40036_MOESM1_ESM.pdf]

Supplementary Materials

For

A renal YY1-KIM1-DR5 axis regulates the progression of acute kidney injury

Chen Yang[1#], Huidie Xu[1#], Dong Yang[1,2], Yunhao Xie[2], Mingrui Xiong[1], Yu Fan[2], XiKai Liu[2], Yu Zhang[1], Yushuo Xiao[1], Yuchen Chen[1], Yihao Zhou[2], Liangliang Song[1], Chen Wang[1], Anlin Peng[3], Robert B. Petersen[4], Hong Chen[1*], Kun Huang[1,5*], Ling Zheng[2*]

[1]School of Pharmacy, Tongji Medical College and State Key Laboratory for Diagnosis and Treatment of Severe Zoonotic Infectious Diseases, Huazhong University of Science and Technology, Wuhan, 430030, China

[2]Hubei Key Laboratory of Cell Homeostasis, Frontier Science Center for Immunology and Metabolism, College of Life Sciences, Wuhan University, Wuhan, 430072, China

[3]Department of Pharmacy, The Third Hospital of Wuhan, Tongren Hospital of Wuhan University, Wuhan, 430070, China

[4]Foundational Sciences, Central Michigan University College of Medicine, Mt. Pleasant, MI, 48859, USA

[5]Tongji-RongCheng Biomedical Center, Tongji Medical College, Huazhong University of Science and Technology, Wuhan, 430030, China

[#] These authors contribute equally

Corresponding authors

Ling Zheng, Ph.D., College of Life Sciences, Wuhan University, Wuhan, China, 430072, lzheng@whu.edu.cn.

Kun Huang, Ph.D., Tongji School of Pharmacy, Huazhong University of Science & Technology, Wuhan, China, 430030, kunhuang@hust.edu.cn.

Hong Chen, Ph.D., Tongji School of Pharmacy, Huazhong University of Science & Technology, Wuhan, China, 430030, hongchen2017@hust.edu.cn.

Supplementary Table of Contents

Supplementary Table 1. ChIP-seq data for KIM1 obtained from GTRD.

Supplementary Table 2. Top 20 KIM1-binding partners.

Supplementary Table 3. Protein-protein interaction (PPI) modeling of human KIM1 and DR5 truncations.

Supplementary Table 4. Potential antagonist peptides that block human KIM1-DR5 interaction designed by Alphafold2 PPI modeling.

Supplementary Table 5. *In silico* calculation of effects of three antagonist peptides on mouse KIM1-DR5 interaction.

Supplementary Table 6. Primers for ChIP assays used in this study.

Supplementary Table 7. Primers for qPCR used in this study

Supplementary Fig. 1. KIM1 is upregulated in renal I/R-induced AKI models.

Supplementary Fig. 2. Expression pattern of YY1 in cisplatin-injured and uIRI-induced mice models and its correlation with KIM1.

Supplementary Fig. 3. Identification of DR5 as a potential binding partner of KIM1.

Supplementary Fig. 4. Nystatin inhibits the formation of high-ordered DR5 oligomers and protects against cisplatin injury in cultured tubular cells.

Supplementary Fig. 5. Validation of $Kim1^{Ksp}$ KO.

Supplementary Fig. 6. Renal tubular specific knockout of Kim1 attenuates inflammation in mouse AKI models.

Supplementary Fig. 7. Screening of peptides blocking KIM1-DR5 interaction by Alphafold2 and their effects against cisplatin injury in cultured renal tubular cells.

Supplementary Fig. 8. *Ex vivo* distribution of Cy7-labeled peptide P2 in cisplatin-injured AKI mice models and co-staining of P2, KIM1, DR5 in HK-2 cells.

Supplementary Table 1. ChIP-seq data for KIM1 obtained from GTRD.

Binding Site	From	To	Length	Sample type	Score
A	157069032	157069520	489	lymphoblastoid cells	6.27541
B	157069668	157070025	358	HEK293T	12
C	157069783	157070140	358	lymphoblastoid cells	6.27541
D	157069983	157070356	374	lymphoblastoid cells	4.53518

Binding site A indicates that YY1 binds with *KIM1* promoter in the P3 region. Binding sites B-D indicate that YY1 binds with *KIM1* promoter in the P4 region.

Supplementary Table 2. Top 20 KIM1-binding partners

Potential KIM1 binding partners	Sum PEP Score	Score Sequest HT	Peptides Sequest HT	Coverage
Transketolase	469.0391571	322.48	37	79.93579454
T-complex protein 1 subunit gamma	432.7576569	263.158	47	80.73394495
Tumor necrosis factor receptor superfamily member 10B	418.3652	293.018	44	72.58704
Stress-70 protein, mitochondrial	415.7114509	299.768	44	65.39027982
Heterogeneous nuclear ribonucleoprotein M	399.5781672	292.527	50	72.87671233
Pyruvate kinase PKM	397.0157855	291.608	41	78.34274953
Stress-induced-phosphoprotein 1	389.859977	230.577	57	75.32228361
X-ray repair crs-complementing protein 6	384.8400658	225.776	48	70.93596059
T-complex protein 1 subunit epsilon	384.7864825	242.817	37	73.56746765
Arginine--tRNA ligase	378.976953	203.943	46	67.12121212
Bifunctional purine biosynthesis protein PURH	371.9582963	203.286	37	75.50675676
Succinate dehydrogenase [ubiquinone] flavoprotein subunit	368.2399038	184.958	33	77.71084337
T-complex protein 1 subunit alpha	357.4008148	210.71	35	81.29496403
T-complex protein 1 subunit zeta	350.0370489	263.084	33	72.12806026
Plastin-3	344.3909043	233.132	42	80.79365079
Lamin-B1	334.6964893	193.145	47	68.94197952
T-complex protein 1 subunit eta	321.4935413	218.57	37	75.69060773
Trifunctional enzyme subunit alpha	315.4693919	158.729	38	66.97247706
Cystathionine beta-synthase-like protein	306.6156272	172.951	26	68.60254083
Tyrosine--tRNA ligase, cytoplasmic OS	305.2868296	178.5064492	45	74.24242424

Supplementary Table 3. Protein-protein interaction (PPI) modeling of human KIM1 and DR5 truncations

PPI	Interface area (Å^2)	$\Delta^i G$ （kcal/mol）
KIM1-DR5	653.8	-5.2
KIM1-DR5 ΔECD	99	-1.2
KIM1-DR5 ΔTMH	591.3	-3.5
KIM1-DR5 ΔCytD	425.0	-3.2

Interface area, accessible surface area; $\Delta^i G$ Solvation energy effect, kcal/mol.

Supplementary Table 4. Potential antagonist peptides that block human KIM1-DR5 interaction designed by Alphafold2 PPI modeling.

	Source	Sequence	Length (AA)	Interface area (Å^2)	Δ^iG (kcal/mol)	$\Delta\Delta^iG$ (kcal/mol)
	34-47	LPCHYSGAVTSMCW	14	789.8	-10.4	-5.2
	60-75	GIVWTNGTHVTYRKDT	16	583.5	0.4	5.6
**KIM1**	103-121	YCCRVEHRGWFNDMKITVS	19	446.2	-4.6	0.6
	302-323	ISVLVLLALLGVIIAKKYFFKK	22	667.7	-5.9	-0.7
	68-85	APQQKRSSPSEGLCPPGH	18	99.3	-0.7	4.5
	102-114	DYSTHWNDLLFCL	13	838.8	-9.7	-4.5
**DR5**	126-140	LSPCTTTRNTVCQCE	15	569.2	1.5	6.7
	217-241	GVTVAAVVLIVAVFVCKSLLWKKVL	25	674.4	-8.2	-3
	344-355	DFADLVPFDSWE	12	516.3	-2.8	2.4
	373-382	AEAAGHRDTL	10	549.1	-5.2	0

Interface area, accessible surface area; Δ^iG Solvation energy effect, kcal/mol; $\Delta\Delta^iG$ =PostΔ^iG of (KIM1-DR5) -PreΔ^iG of (KIM1-DR5).

The Δ^iG of human (KIM1-DR5) is -5.2 kcal/mol.

Supplementary Table 5. ***In silico*** **calculation of effects of three antagonist peptides on mouse KIM1-DR5 interaction.**

Blocking Peptides	Source	Sequence	Length (AA)	Interface area (Å^2)	$\Delta^i G$ (kcal/mol)	$\Delta\Delta^i G$ (kcal/mol)
**P1**	KIM1 60-75	GIVWTNGTHVTYRKDT	16	129.1	-2.7	3.8
**P2**	DR5 68-85	APQQKRSSPSEGLCPPGH	18	153.0	0.1	6.6
**P3**	DR5 126-140	LSPCTTTRNTVCQCE	15	107.8	-1.4	5.1

Interface area, accessible surface area; $\Delta^i G$ Solvation energy effect, kcal/mol; $\Delta\Delta^i G$ = Post$\Delta^i G$ of (KIM1-DR5) - Pre$\Delta^i G$ of (KIM1-DR5). The $\Delta^i G$ of mouse (KIM1-DR5) is -6.5 kcal/mol.

Supplementary Table 6. Primers for ChIP assays used in this study.

Gene			Sequences
Human *KIM1*	P1	Forward	GCTGTCCAGAAGAGGGGAATC
		Reverse	CTCACTTTGTTTCCCCACGGA
	P2	Forward	CACGTGTGTCATGGTGCTAAG
		Reverse	GACTTTTCCCTACTCCCGCC
	P3	Forward	TGTTTTGGAAAGGCAAAGTGTCT
		Reverse	ACCCAGCCTTAACTGTTCATGT
	P4	Forward	GGGCAACAGGATGCTTGCTT
		Reverse	TGGCCACATTTGGGAAAACTGA
Mouse *Kim1*	P1	Forward	GCCCTTTATGGCGAAGTCCA
		Reverse	AGAGGAAGCCAGCTGAACAC
	P2	Forward	GAAGGCTCTGCCATTTCAGC
		Reverse	GTTGAACGGCCCTTAAGCAG
	P3	Forward	GATGGTCTCACCCACAAGGG
		Reverse	TGCCTCCATGAGATCCAACTG
	P4	Forward	CTCCTCTTCGAAACCTCTCCC
		Reverse	AATAGAACCCAGTCAGGCTCG

Supplementary Table 7. Primers for qPCR used in this study.

	Gene	Forward	Reverse
**Human**	*KIM1*	TGGCAGATTCTGTAGCTGGTT	AGAGAACATGAGCCTCTATTCCA
	IL6	ACTCACCTCTTCAGAACGAATTG	CCATCTTTGGAAGGTTCAGGTTG
	CXCL2	CAAGAACATCCAAAGTGTGA	CCATTCTTGAGTGTGGCTAT
	CXCL10	GTGGCATTCAAGGAGTACCTC	TGATGGCCTTCGATTCTGGATT
	YY1	ACGGCTTCGAGGATCAGATTC	TGACCAGCGTTTGTTCAATGT
	FAS	TCTGGTTCTTACGTCTGTTGC	CTGTGCAGTCCCTAGCTTTCC
	BAX	CCCGAGAGGTCTTTTTCCGAG	CCAGCCCATGATGGTTCTGAT
	BCL2	GGTGGGGTCATGTGTGTGG	CGGTTCAGGTACTCAGTCATCC
	CYR61	TAAGGTCTGCGCTAAACAACTC	CAGATCCCTTTCAGAGCGGT
**Mouse**	*Nhe3*	CAAGGTCACCAGTATCGTCCC	GCATGAAGTATCCAGCATCCAAC
	Fabp4	AAGGTGAAGAGCATCATAACCCT	TCACGCCTTTCATAACACATTCC
	Il18	GTGAACCCCAGACCAGACTG	CCTGGAACACGTTTCTGAAAGA
	Kim1	ACATATCGTGGAATCACAACGAC	ACTGCTCTTCTGATAGGTGACA
	Ngal	TGGCCCTGAGTGTCATGTG	CTCTTGTAGCTCATAGATGGTGC
	Yy1	GTGGTTGAAGAGCAGATCATTGG	TTGCTTAGGGTCTGAGAGGTC
	Esr1	CCCGCCTTCTACAGGTCTAAT	CTTTCTCGTTACTGCTGGACAG
	Usf1	CTGAAACCGAAGAGGGAACAG	GTTGGGGTCAGGAAAAGTGG
	Esr2	CTGTGATGAACTACAGTGTTCCC	CACATTTGGGCTTGCAGTCTG

	Erg	CCAGCAGCTCATATTAAGGAGG	CGTTCCGTAGGCACACTCA
	Ctcf	CAGTGGACGATACCCAGATCA	CCTTCAGGCAAAGGTAAGGTG
	Cdx2	TACCCGGACTACGGTGGTTAC	GTGATGGTGCGCGTGGTAT
	Gata4	CACCCCAATCTCGATATGTTTGA	GCACAGGTAGTGTCCCGTC
	Cebpa	GCGGGAACGCAACAACATC	GTCACTGGTCAACTCCAGCAC
	Nr3c1	GACTCCAAAGAATCCTTAGCTCC	CTCCACCCCTCAGGGTTTTAT
	Foxa1	ACATTCAAGCGCAGCTACCC	TGCTGGTTCTGGCGGTAATAG
	Gata6	TTGCTCCGGTAACAGCAGTG	GTGGTCGCTTGTGTAGAAGGA
	Foxa2	TCCGACTGGAGCAGCTACTAC	GCGCCCACATAGGATGACA
	Gata3	AAGCTCAGTATCCGCTGACG	GTTTCCGTAGTAGGACGGGAC
	Tcf7l2	TCATCACGTACAGCAATGAACA	CGACAGCGGGTAATATGGAGAG
**Mouse**	*Tcf12*	ATGTACTGTGCTTATCCTGTCCC	GGTGCATATACCGTTTTCCCATT
	Fosl2	CACGCCGAGTCCTACTCCA	GTGGGCTGTACCATCCACTG
	Stat1	TCACAGTGGTTCGAGCTTCAG	CGAGACATCATAGGCAGCGTG
	Stat3	CACCTTGGATTGAGAGTCAAGAC	AGGAATCGGCTATATTGCTGGT
	Cebpb	GGTGCCCGCTGCAGTTT	CTCGCAGTTTAGTGGTGGTAAGTC
	Max	CAAGCGGGCTCACCATAATG	TGTTGCTTTGTCTAGGATTTGGG
	Spi1	TTACAGGCGTGCAAAATGGAA	GACGTTGGTATAGCTCTGAATCG
	Sp1	AGGGTCCGAGTCAGTCAGG	CTCGCTGCCATTGGTACTGTT
	Il6	TCTGCAAGAGACTTCCATCCAGTTGC	AGCCTCCGACTTGTGAAGTGGT
	Fas	GCGGGTTCGTGAAACTGATAA	GCAAAATGGGCCTCCTTGATA
	Bax	AGACAGGGGCCTTTTTGCTAC	AATTCGCCGGAGACACTCG

Mouse	*Bcl2*	GCTACCGTCGTGACTTCGC	CCCCACCGAACTCAAAGAAGG
	Tnfa	GGTGATCGGTCCCCAAAGGGATGA	TGGTTTGCTACGACGTGGGCT
	Il1b	AAAGCCTCGTGCTGTCGGACC	CAGGGTGGGTGTGCCGTCTT
	Mcp1	TAAAAACCTGGATCGGAACCAAA	GCATTAGCTTCAGATTTACGGGT
	Cxcl2	ACGGAAGAACCAAAGAGAA	AAATAAGTGAACTCTCAGACAGC
	Cxcl10	ATCATCCCTGCGAGCCTATCCT	GACCTTTTTTGGCTAAACGCTTTC
	Tgfb1	GACTCTCCACCTGCAAGACC	GGACTGGCGAGCCTTAGTTT
	Fibronectin	ATGTGGACCCCTCCTGATAGT	GCCCAGTGATTTCAGCAAAGG
	Col1a1	GCTCCTCTTAGGGGCCACT	ATTGGGGACCCTTAGGCCAT
	Ksp-Cre	GCAGATCTGGCTCTCCAAAG	AGGCAAATTTTGGTGTACGG
	Flox/Flox	ACTTGGTCCCATGTGAGAACATG	AAGATGCTAAGCCTTCGTTGACC

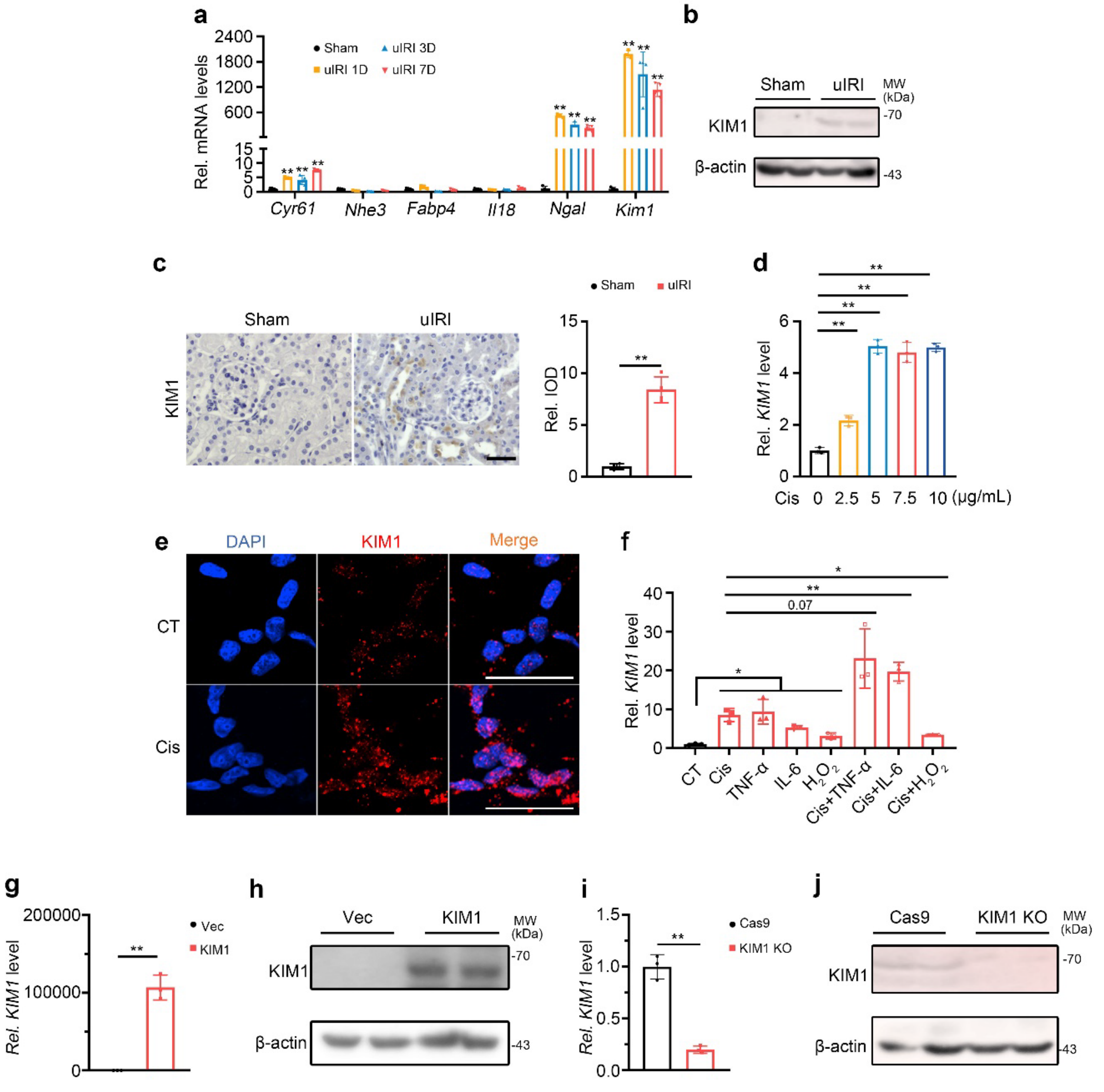

Supplementary Fig 1. KIM1 is dramatically upregulated in unilateral renal ischemia-reperfusion injured mice. a qPCR analysis of several known AKI biomarkers in mouse kidneys at indicated time after unilateral renal ischemia-reperfusion injury (uIRI). Sham, non-injury control; uIRI, unilateral ischemia reperfusion injury. n = 4 mice per group. **b** Western blot analysis of KIM1 protein level in mouse kidneys at Day 3 after uIRI. n = 2 mice per group, each experiment was repeated at least three times independently with

similar results obtained. **c** Representative images of KIM1 immunohistochemistry staining with quantitative analysis at Day 3 after uIRI. Scale bar, 50 μm. n = 4 mice per group. **d** qPCR analysis of *KIM1* level at indicated dosage in 24 hrs cisplatin-injured HK-2 cells. n = 3 biological samples per group. **e** Representative immunofluorescence staining of KIM1 at 24 hrs of 5 μg/mL cisplatin injured HK-2 cells. Scale bar, 50 μm. Each experiment was repeated at least three times independently with similar results obtained. **f** qPCR analysis of *KIM1* level in HK-2 cells with indicated stimuli with or without 5 μg/mL cisplatin. IL-6 (50 ng/mL), TNF-α (20 ng/mL), H_2O_2 (800 μM). n = 3 biological samples for per group. **g-j** Efficiency of KIM1 overexpression (g-h) or knockout (i-j) in HK-2 cells. (g & I) n = 3 biological samples per group; (h & j) each experiment was repeated at least three times independently with similar results obtained. Vec, pRK-5'Flag; KIM1, pRK-5'Flag-KIM1; Cas9, lenti-CRISPR/Cas9; KIM1 KO, lenti-CRISPR/Cas9-based KIM1 knockout. Data were shown as mean ± SD. Two-tailed unpaired Student's t test was used for two experimental groups, and one-way ANOVA for multiple experimental groups without adjustment. $^{*}P < 0.05$; $^{**}P < 0.01$. Exact *P* values are provided in Source Data file.

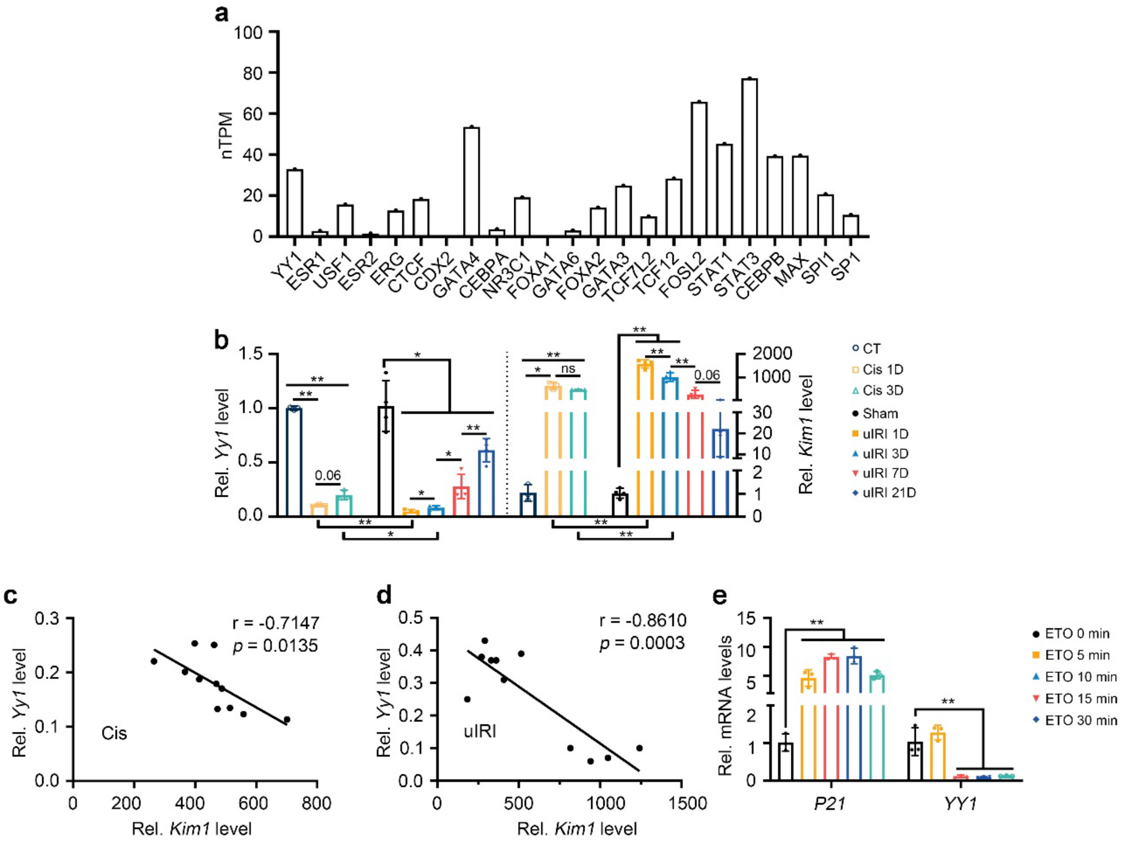

Supplementary Fig 2. Expression pattern of YY1 in cisplatin-injured and uIRI mice models and its correlation with KIM1. a Expression levels of 23 transcription factors in the kidney as indicated by the Human Protein ATLAS dataset (HPA). nTPM, number of protein-coding transcripts per million. **b** Expression pattern of *YY1* and *KIM1* in the kidneys of cisplatin (Cis)- or unilateral renal ischemia-reperfusion injury (uIRI)-injured mice for indicated time. Sham, non-injury control; uIRI, unilateral ischemia reperfusion injury. n = 4 mice per group; two-tailed unpaired Student's t test was used. **c-d** Correlation analysis of *KIM1* and *YY1* mRNA levels at Day 3 after cisplatin injury (c) or uIRI (d). n = 11 mice for CT and Cis 3D groups; n = 12 mice for sham and uIRI 3D groups. **e** qPCR analysis of *p21* and *YY1* levels in HK-2 cells treated with 50 μM etoposide (ETO) for

indicated time. n = 3 biological samples per group. Data are shown as mean ± SD. Two-tailed unpaired Student's t test was used for two experimental groups, and one-way ANOVA for multiple experimental groups without adjustment. $^{*}P < 0.05$; $^{**}P < 0.01$; ns, no significance. Exact *P* values are provided in Source Data file.

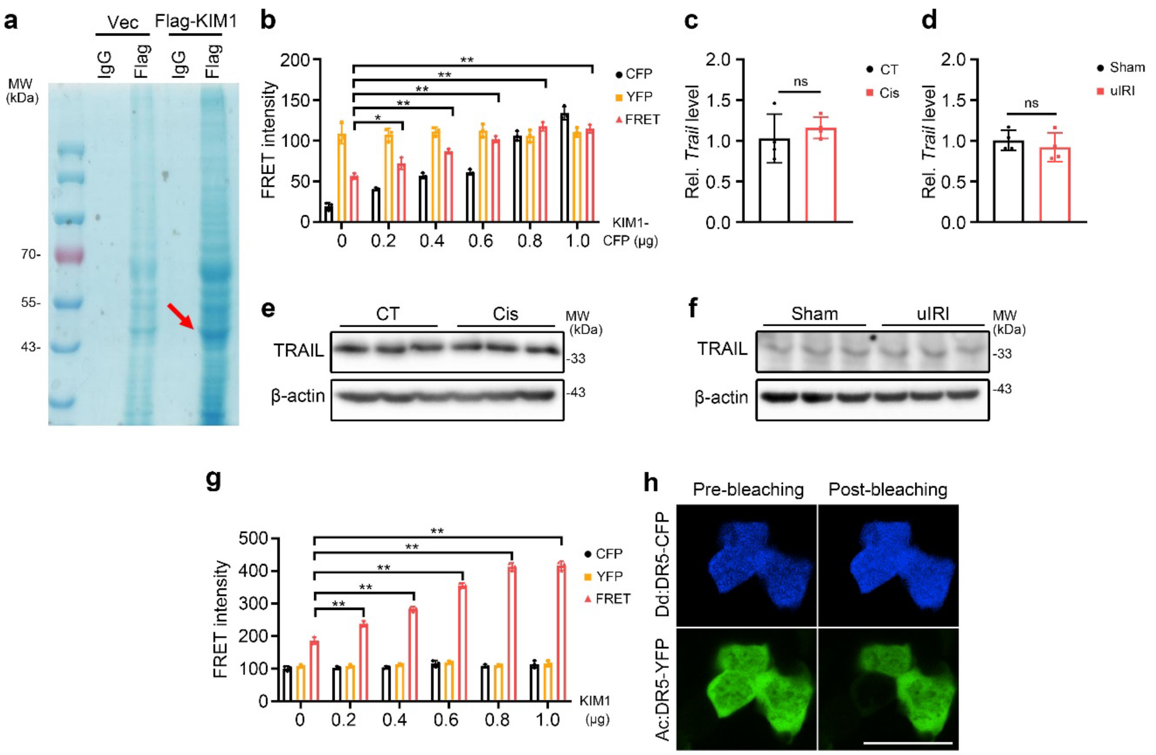

Supplementary Fig 3. Identification of DR5 as a potential binding partner of KIM1.

a SDS-PAGE of *KIM1*-overexpressed HEK293T cells, and Flag-pull down sample was used for mass spectrometry detection. Red arrow indicates DR5 containing band. Flag-KIM1, pRK-5'Flag-KIM1. Each experiment was repeated at least three times independently with similar results obtained. **b** Effects of gradient KIM1-CFP overexpression on the FRET intensity between KIM1-CFP and DR5-YFP in HK-2 cells. KIM1-CFP, pRK-5'Flag-KIM1-CFP. n = 3 biological samples per group. **c-f** qPCR (c-d) and Western blot analysis (e-f) of TRAIL at Day 3 after cisplatin injury (c & e) or unilateral renal ischemia-reperfusion injury (uIRI, d & f). (c-d) n = 4 mice per group; (e-f) n = 3 mice per group. **g** FRET analysis showed the effects of gradient KIM1 overexpression on DR5-CFP/DR5-YFP multimerization in HK-2 cells. n = 3 biological samples per group. **h**

Representative images of acceptor photobleaching analysis of DR5-CFP and DR5-YFP. Photobleaching of receptor (DR5-YFP) led to enhanced acceptor fluorescence (DR5-CFP). n = 3 biological samples per group. Data are shown as mean ± SD. Two-tailed unpaired Student's t test was used for two experimental groups, and one-way ANOVA for multiple experimental groups without adjustment. Scale bar, 25 μm. *$P < 0.05$; **$P < 0.01$; ns, no significance. Exact P values are provided in Source Data file.

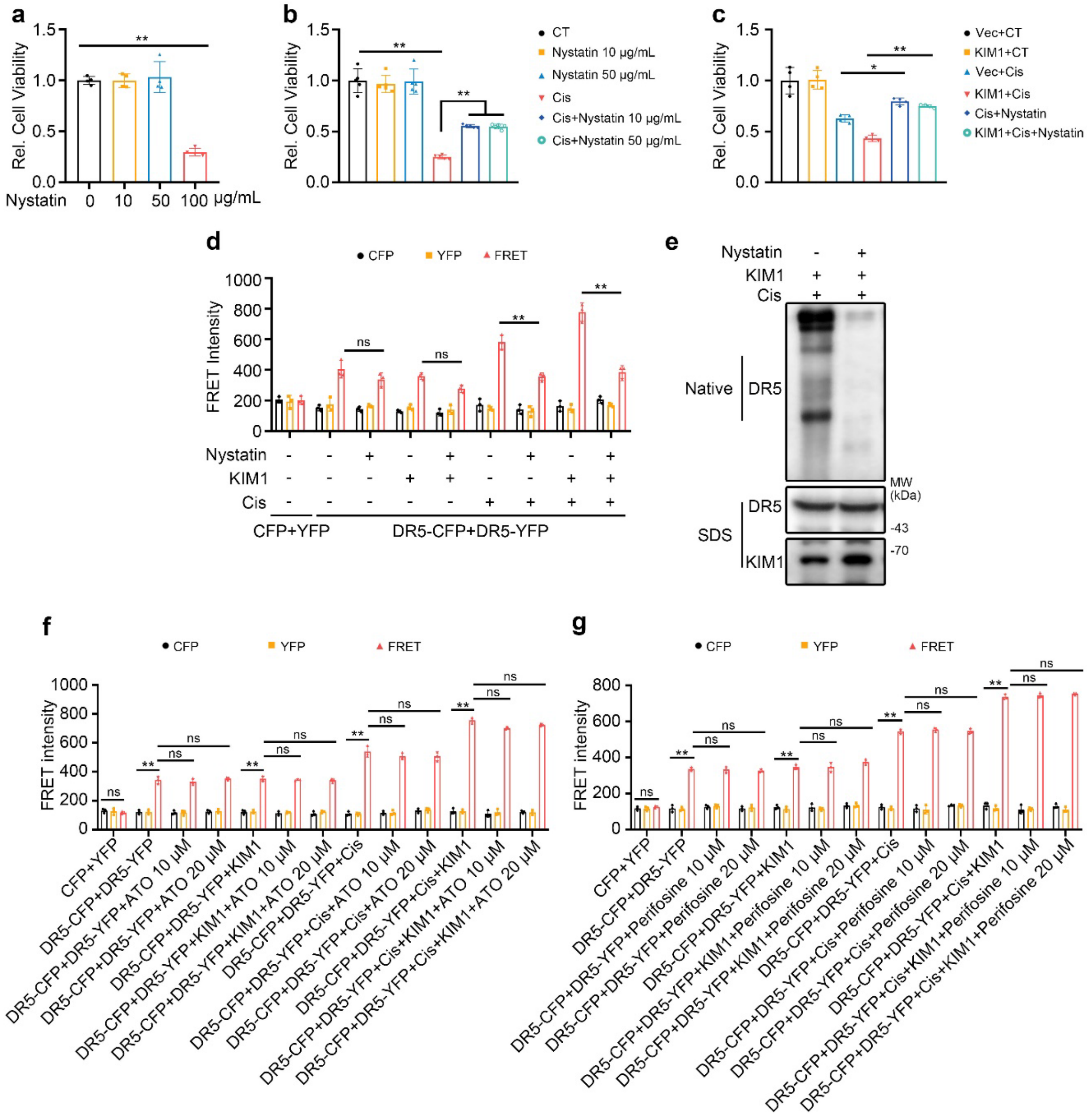

Supplementary Fig 4. Nystatin inhibits the formation of higher-order DR5 oligomers and protects against cisplatin injury in cultured renal tubular cells. a MTT assays showed the effect of indicated dosage of nystatin on HK-2 cells. n = 5 biological samples per group, each experiment was repeated at least three times independently with similar results obtained. **b** MTT assays showed the effects of indicated dosage of nystatin on cisplatin injury induced cell death (Cis, 5 μg/mL, 24 hrs) in HK-2 cells. n = 5 biological

samples per group, each experiment was repeated at least three times independently with similar results obtained. **c** MTT assays showed the effects of 10 μg/mL nystatin on cisplatin injury induced cell death with or without KIM1 overexpression in HK-2 cells. n = 4 biological samples per group, each experiment was repeated at least three times independently with similar results obtained. **d** FRET analysis showed the effects of 10 μg/mL nystatin on DR5 multimerization in HK-2 cells with/without cisplatin injury. CFP, pRK-5'Flag-CFP; YFP, pRK-5'Flag-YFP; DR5-CFP, pRK-5'Flag-DR5-CFP; DR5-YFP, pRK-5'Flag-DR5-YFP; n = 3 biological samples per group. **e** Native PAGE showed DR5 multimerization in the presence/absence of 10 μg/mL nystatin with KIM1 overexpression under cisplatin injury. Each experiment was repeated at least three times independently with similar results obtained. **f-g** FRET analysis showed the effects of atorvastatin (ATO) (f) and perifosine (g) on DR5 multimerization in HK-2 cells with or without cisplatin injury. n = 3 biological samples per group, each experiment was repeated at least three times independently with similar results obtained. Data are shown as mean ± SD. Two-tailed unpaired Student's t test was used for two experimental groups, and one-way ANOVA for multiple experimental groups without adjustment. **P* < 0.05; ***P* < 0.01; ns, no significance. Exact *P* values are provided in Source Data file.

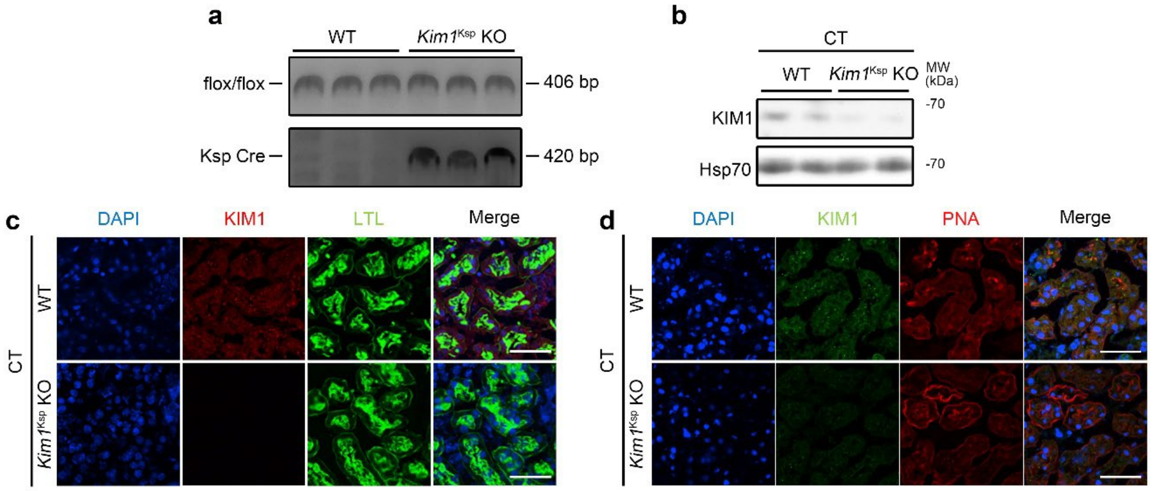

Supplementary Fig 5. Validation of *Kim1*$^{\mathbf{Ksp}}$ KO mouse. a Genotyping results. n = 3 individual mice for WT and *Kim1*^{Ksp} KO groups. **b** Western blot analysis of KIM1 protein levels in the kidneys of WT and *Kim1*^{Ksp} KO mice under normal conditions (CT). n = 2 mice per group, each experiment was repeated at least three times independently with similar results obtained. **c** Representative images of Kim1 (red) with LTL (lotus tetragonolobus lectin, green) in the kidneys of WT and *Kim1*^{Ksp} KO mice under normal conditions. Nuclei were stained with DAPI (blue). Scale bar, 50 µm. n = 3 mice per group. **d** Representative images of Kim1 (green) with PNA (peanut agglutinin, red) of WT and *Kim1*^{Ksp} KO mice under normal conditions. Nuclei were stained with DAPI (blue). n = 3 mice per group. Scale bar, 50 µm.

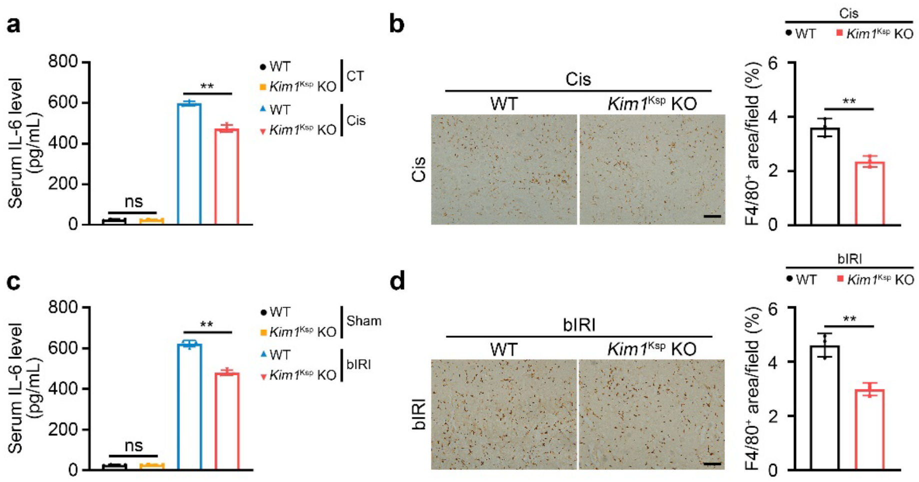

Supplementary Fig 6. Renal tubular specific knockout of *Kim1* attenuates inflammation in mouse AKI models. a & c Serum IL-6 levels in WT and *Kim1*^{Ksp} KO mice at Day 3 after cisplatin injury (Cis, a) or at Day 1 after bilateral renal ischemia-reperfusion injury (bIRI, c). **b & d** IHC staining of F4/80 with quantitative results in WT and *Kim1*^{Ksp} KO mice at Day 3 after cisplatin injury (b) or at Day 1 after bIRI (d). Scale bar, 50 µm. (a-d) n = 3 mice per group. Data are shown as mean ± SD. Two-tailed unpaired Student's t test was used for two experimental groups, and one-way ANOVA for multiple experimental groups without adjustment. $^{**}P < 0.01$; ns, no significance. Exact *P* values are provided in Source Data file.

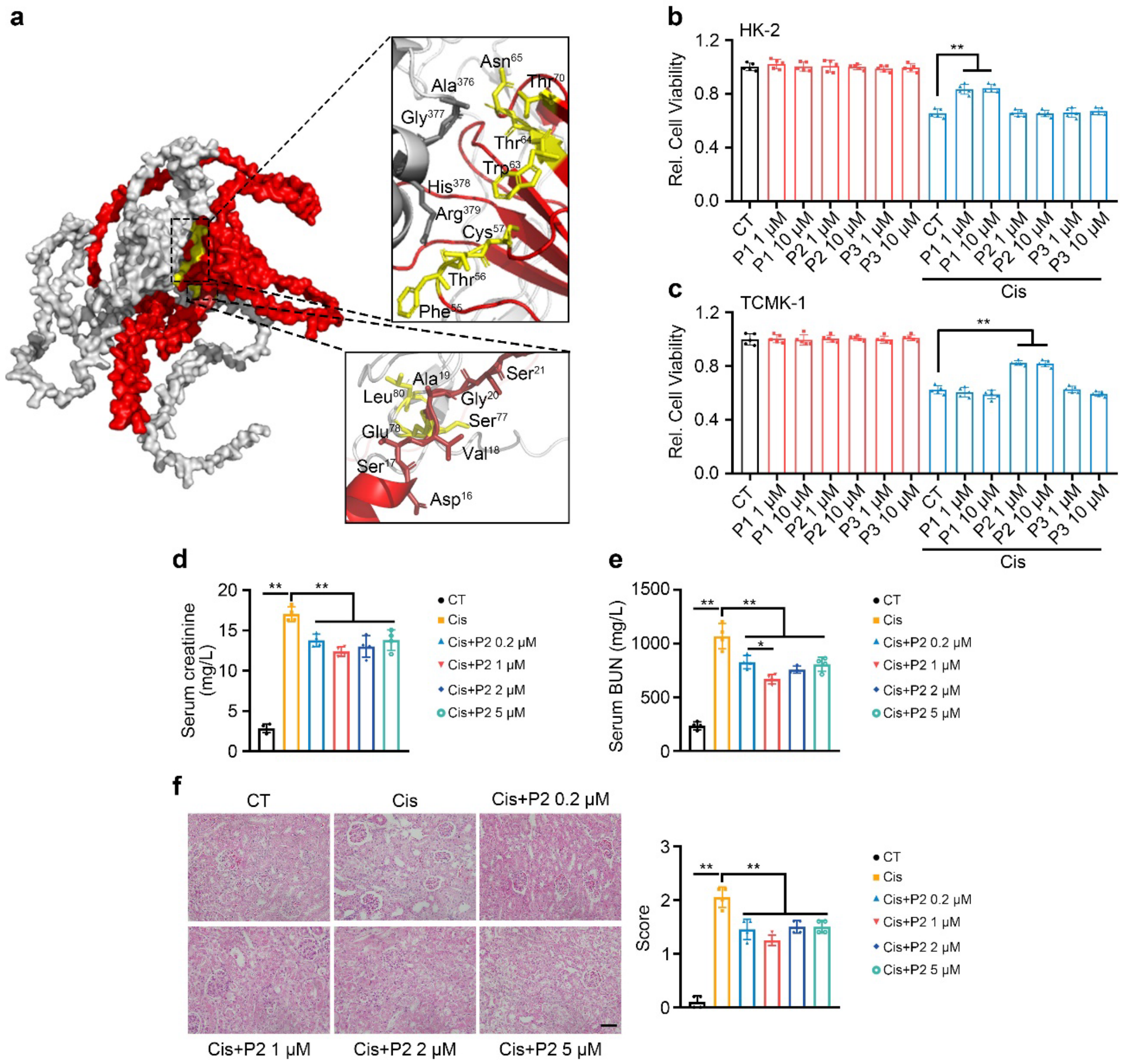

Supplementary Fig 7. Screening of peptides blocking KIM1-DR5 interaction by Alphafold2 and their effects against cisplatin injury in cultured renal tubular cells. a A simulated low-energy binding conformation of KIM1-DR5 complex. **b-c** Effects of three antagonistic peptides (P1-P3) against 24 hrs cisplatin-injury in human HK-2 (b) and mouse TCMK-1 (c) cells. n = 5 biological samples per group, each experiment was repeated at least three times independently with similar results obtained. **d-e** Serum creatinine (d) and urea nitrogen levels (e) in cisplatin-injured mice treated with different dosages of peptide

P2. n = 4 mice per group. **f** Representative H&E staining and pathological score in cisplatin-injured mice treated with different dosages of peptide P2. Scale bar, 50 μm. n = 4 mice per group. $^{*}P < 0.05$; $^{**}P < 0.01$. Exact *P* values are provided in Source Data file.

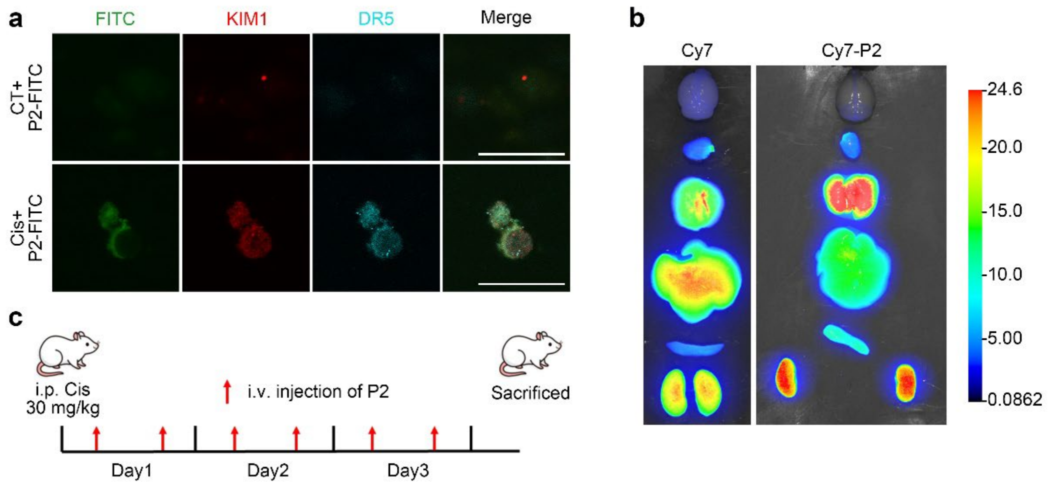

Supplementary Fig 8. ***Ex vivo*** **distribution of Cy7-labeled peptide P2 in cisplatin-injured AKI mice models and co-staining of P2, KIM1, DR5 in HK-2 cells. a** Representative image of P2-FITC (green), KIM1(red) and DR5 (cyan) in HK-2 cells with or without 24 hrs cisplatin injury. Scale bar, 50 μm. Each experiment was repeated at least three times independently with similar results obtained. **b** *Ex vivo* distribution of Cy7-labeled peptide P2 (Cy7-P2) vs Cy7 *per se* in cisplatin-injured mouse. Top to bottom: brain, heart, lung, liver, spleen and kidney. **c** Schematic diagram of peptide P2 treatment in cisplatin (Cis)-injured mice. i.p., intraperitoneal injection; i.v., intravenous injection.
