# Supplementary material for: A renal YY1-KIM1-DR5 axis regulates the progression of acute kidney injury
Source: Nat Commun. 2023 Jul 17;14:4261. doi: 10.1038/s41467-023-40036-z (PMC10352345; doi:10.1038/s41467-023-40036-z)
Supplement: Supplementary file 3 — Reporting Summary [file 41467_2023_40036_MOESM3_ESM.pdf]

nature portfolio

Corresponding author(s): Ling Zheng, Kun Huang, Hong Chen

Last updated by author(s): Jun 23, 2023

Reporting Summary

Nature Portfolio wishes to improve the reproducibility of the work that we publish. This form provides structure for consistency and transparency in reporting. For further information on Nature Portfolio policies, see our Editorial Policies and the Editorial Policy Checklist.

Statistics

For all statistical analyses, confirm that the following items are present in the figure legend, table legend, main text, or Methods section.

n/a	Confirmed	
☐	☒	The exact sample size (*n*) for each experimental group/condition, given as a discrete number and unit of measurement
☐	☒	A statement on whether measurements were taken from distinct samples or whether the same sample was measured repeatedly
☐	☒	The statistical test(s) used AND whether they are one- or two-sided<br>*Only common tests should be described solely by name; describe more complex techniques in the Methods section.*
☒	☐	A description of all covariates tested
☒	☐	A description of any assumptions or corrections, such as tests of normality and adjustment for multiple comparisons
☐	☒	A full description of the statistical parameters including central tendency (e.g. means) or other basic estimates (e.g. regression coefficient) AND variation (e.g. standard deviation) or associated estimates of uncertainty (e.g. confidence intervals)
☐	☒	For null hypothesis testing, the test statistic (e.g. *F*, *t*, *r*) with confidence intervals, effect sizes, degrees of freedom and *P* value noted<br>*Give P values as exact values whenever suitable.*
☒	☐	For Bayesian analysis, information on the choice of priors and Markov chain Monte Carlo settings
☒	☐	For hierarchical and complex designs, identification of the appropriate level for tests and full reporting of outcomes
☐	☒	Estimates of effect sizes (e.g. Cohen's *d*, Pearson's *r*), indicating how they were calculated

Our web collection on statistics for biologists contains articles on many of the points above.

Software and code

Policy information about availability of computer code

Data collection	Images for western blots were taken by KwikQuant Imager (Kindle Biosciences, USA).<br>Images were taken by BX60 Microscope (Olympus, Japan) or RX50 Microscope (Sunny Optical Technology, China).<br>Confocal images were taken by AX confocal microscope (NIKON, Japan).<br>Quantitative real-time PCR were taken by Bio-Rad CFX 96 (Bio-Rad, USA).
Data analysis	The following softwares were used for data analysis:<br>Relative integrated option density (IOD) was calculated using Image J 1.53 (USA) as described.<br>Protein-protein interaction was visualized with Pymol 2.3.0 (USA).<br>Data were analyzed with GraphPad Prism 8.0 (USA).

For manuscripts utilizing custom algorithms or software that are central to the research but not yet described in published literature, software must be made available to editors and reviewers. We strongly encourage code deposition in a community repository (e.g. GitHub). See the Nature Portfolio guidelines for submitting code & software for further information.

Data

Policy information about availability of data

All manuscripts must include a data availability statement. This statement should provide the following information, where applicable:

- Accession codes, unique identifiers, or web links for publicly available datasets
- A description of any restrictions on data availability
- For clinical datasets or third party data, please ensure that the statement adheres to our policy

Source data is provided with this paper for Figures 1-8, Supplementary Figures 1-7. Databases used in this study include Human Protein Atlas (https://www.proteinatlas.org), JASPAR (https://jaspar.genereg.net), hTFtarget (http://bioinfo.life.hust.edu.cn/hTFtarget), Human TFBD 3.0 (http://bioinfo.life.hust.edu.cn/HumanTFDB), and GTRD (http://gtrd.biouml.org).

Research involving human participants, their data, or biological material

Policy information about studies with human participants or human data. See also policy information about sex, gender (identity/presentation), and sexual orientation and race, ethnicity and racism.

Reporting on sex and gender	N/A
Reporting on race, ethnicity, or other socially relevant groupings	N/A
Population characteristics	N/A
Recruitment	N/A
Ethics oversight	N/A

Note that full information on the approval of the study protocol must also be provided in the manuscript.

Field-specific reporting

Please select the one below that is the best fit for your research. If you are not sure, read the appropriate sections before making your selection.

☒ Life sciences ☐ Behavioural & social sciences ☐ Ecological, evolutionary & environmental sciences

For a reference copy of the document with all sections, see nature.com/documents/nr-reporting-summary-flat.pdf

Life sciences study design

All studies must disclose on these points even when the disclosure is negative.

Sample size	All relevant sample sizes are described in the legend to each figure and/or in the methods section. Sample sizes were determined based on previous experience (DOI: 10.1038/s41467-022-31476-0; DOI: 10.7150/thno.72515; DOI: 10.15252/embr.202256128), literature standards, or pilot data to ensure the possibility of statistical analysis and to minimize the use of experimental animals based on the 3R principles. Additional details regarding sample size are described in figure legends and can be found in Source Data.
Data exclusions	No data was excluded from analysis.
Replication	All experiments using animals were performed with at least three biological replicates; all experiments using cultured cells were independently performed for three times and similar results were obtained. Relevant information of replication was mentioned in indicated figure legends.
Randomization	Mice and cells in a given experiment were randomly assigned to different groups.
Blinding	Blinding was done for staining analysis. For the other animal studies and cell cultures, blinding was not done since the experimental design requires the investigators to know genotype information for each mouse or the treatment for cells. Most of the experiments were designed, performed and analyzed by the same person. However, no data was excluded in this study, all results were analyzed in unbiased ways.

Reporting for specific materials, systems and methods

We require information from authors about some types of materials, experimental systems and methods used in many studies. Here, indicate whether each material, system or method listed is relevant to your study. If you are not sure if a list item applies to your research, read the appropriate section before selecting a response.

Materials & experimental systems

n/a	Involved in the study
☐	☒ Antibodies
☐	☒ Eukaryotic cell lines
☒	☐ Palaeontology and archaeology
☐	☒ Animals and other organisms
☒	☐ Clinical data
☒	☐ Dual use research of concern
☒	☐ Plants

Methods

n/a	Involved in the study
☒	☐ ChIP-seq
☐	☒ Flow cytometry
☒	☐ MRI-based neuroimaging

Antibodies

Antibodies used

All antibodies used in this study are listed in methods.
Primary antibodies:
Mouse monoclonal anti-Hsp70 (BD, Cat: 610607) dilution 1:5000 for WB
Mouse monoclonal anti-β-actin (Sigma Aldrich, Cat: A5316, clone: AC-74) dilution 1:10000 for WB
Mouse monoclonal anti-Flag (Sigma Aldrich, Cat: F1804, clone: M2) dilution 1:5000 for WB
Mouse monoclonal anti-HA (Sigma Aldrich, Cat: H3663, clone: HA-7) dilution 1:5000 for WB
Rabbit polyclonal anti-KIM1 (KIM1 was prepared by DaiAn Biotech, Wuhan, China) dilution 1:1000 for WB, dilution 1:200 for IHC/IF
Rabbit monoclonal anti-YY1 (Cell Signaling Technology, Cat: 46395, clone: D5D9Z) dilution 1:1000 for WB, dilution 1:200 for IHC
Rabbit monoclonal anti-DR5 (Cell Signaling Technology, Cat: 8074, clone: D4E9) dilution 1:1000 for WB, dilution 1:200 for IHC/IF
Mouse monoclonal anti-DR5 (Santa Cruz, Cat: Sc-166624, clone: D-6) dilution 1:200 for IF
Rabbit Polyclonal anti-Caspase3 (ABclonal, Cat: A2156) dilution 1:1000 for WB
Rabbit polyclonal anti-c-Caspase3 (Cell Signaling Technology, Cat: 9661) dilution 1:1000 for WB
Rabbit polyclonal anti-Caspase8 (Proteintech, Cat: 13423-1-AP) dilution 1:1000 for WB
Rabbit monoclonal anti-Caspase9 (Abcam, Cat: ab202068, clone: EPR18107) dilution 1:1000 for WB
Rabbit monoclonal anti-PARP-1 (Cell Signaling Technology, Cat: 9532, clone: 46D11) dilution 1:1000 for WB
Rabbit polyclonal anti-p-p53 (Cell Signaling Technology, Cat: 9284) dilution 1:1000 for WB
Rabbit monoclonal anti-F4/80 (Cell Signaling Technology, Cat: 70076, clone: D2S9R) dilution 1:100 for immunostaining
Rabbit polyclonal anti-TRAIL (Proteintech, Cat: 27064-1-AP) dilution 1:1000 for WB

Secondary antibodies:
Goat Anti-Rabbit IgG (H + L)-HRP Conjugate (Biorad, Cat: 1706515) dilution 1:5000 for WB
Goat Anti-Mouse IgG (H + L)-HRP Conjugate (Biorad, Cat: 1706516) dilution 1:5000 for WB
Anti-Rabbit IgG HRP (Rockland, Cat: 18-8816-33) dilution 1:1000 for IP
Anti-Mouse IgG HRP (Rockland, Cat: 18-8817-33) dilution 1:1000 for IP
Goat Anti-Rabbit IgG Antibody (H+L) (Vector, Cat: BA-1000-1.5) dilution 1:500 for IHC
Goat anti-Rabbit IgG (H+L) Cross-Adsorbed Secondary Antibody, Alexa Fluor™ 594 (Invitrogen, Cat: A11012.) dilution 1:500 for IF
Goat anti-Mouse IgG (H+L) Cross-Adsorbed Secondary Antibody, Alexa Fluor™ 488 (Invitrogen, Cat: A-11001.) dilution 1:500 for IF
Goat anti-Mouse IgG (H+L) Cross-Adsorbed Secondary Antibody, Alexa Fluor™ 405(Invitrogen, Cat: A-31553.) dilution 1:500 for IF

Validation

All antibodies used in this study are from commercial suppliers that have verified the specificity of the antibodies using recombinant proteins or knock-out cell lines. Their validation statements are available on the manufacturer's website.
anti-Hsp70 (BD, Cat: 610607) (Species reactivity: Human. Application: WB, IF, IP, IHC.)
https://www.bdbiosciences.com/zh-cn/products/reagents/microscopy-imaging-reagents/immunofluorescence-reagents/purified-mouse-anti-hsp70.610607
anti-β-actin (Sigma Aldrich, Cat: A5316, clone: AC-74) (Species reactivity: rabbit, sheep, cat, guinea pig, bovine, mouse, chicken, wide range, Hirudo medicinalis, canine, Drosophila, carp, pig, rat, human. Application: ELISA (i), IF, IHC (p), WB.)
https://www.sigmaaldrich.cn/CN/zh/product/sigma/a5316
anti-Flag (Sigma Aldrich, Cat: F1804, clone: M2) (Species reactivity: all. Application: WB, IP, IHC, IF, EIA.)
https://www.sigmaaldrich.cn/CN/zh/product/sigma/f1804
anti-HA (Sigma Aldrich, Cat: H3663, clone: HA-7) (Application: ICC, IP, WB.)
https://www.sigmaaldrich.cn/CN/zh/product/sigma/h3663
anti-KIM1 (Species reactivity: Human, Mouse. Application: WB, IP, IHC, IF.)
anti-YY1 (Cell Signaling Technology, Cat: 46395, clone: D5D9Z) (Species reactivity: Human, Mouse, Rat, Monkey. Application: WB, IP, IF-IC, FC-FP, ChIP, ChIP-seq, C&R.)
https://www.cellsignal.cn/products/primary-antibodies/yy1-d5d9z-rabbit-mab/46395?site-search-type=Products&N=4294956287&Ntt=46395&fromPage=plp&_requestid=1008089
anti-DR5 (Cell Signaling Technology, Cat: 8074, clone: D4E9) (Species reactivity: Human. Application: WB, IP, IF-IC.)
https://www.cellsignal.cn/products/primary-antibodies/dr5-d4e9-xp-rabbit-mab/8074?site-search-type=Products&N=4294956287&Ntt=8074&fromPage=plp&_requestid=1005357
anti-DR5 (Santa Cruz, Cat: Sc-166624, clone: D-6) (Species reactivity: Human. Application: WB, IP, IF, ELISA.)
https://www.scbt.com/p/dr5-antibody-d-6?requestFrom=search
anti-Caspase3 (ABclonal, Cat: A2156) (Species reactivity: Human, Mouse, Rat. Application: WB,IHC-P,IF/ICC.)
https://abclonal.com.cn/catalog/A2156
anti-c-Caspase3 (Cell Signaling Technology, Cat: 9661) (Species reactivity: Human, Mouse, Rat, Monkey. Application: WB, W-S, IP, IHC-P, IF-IC, FC-FP.)
https://www.cellsignal.cn/products/primary-antibodies/cleaved-caspase-3-asp175-antibody/9661?site-search-type=Products&N=4294956287&Ntt=9661&fromPage=plp&_requestid=1005733
anti-Caspase8 (Proteintech, Cat: 13423-1-AP) (Species reactivity: Human, Mouse, Rat, Chicken, Pig, Rabbit. Application: ELISA, IF, IHC,

IP, RIP, WB.)
https://www.ptgcn.com/products/CASP8-Antibody-13423-1-AP.htm
anti-Caspase9 (Abcam, Cat: ab202068, clone: EPR18107) (Species reactivity: Mouse, Human. Application: IHC-P, WB, ICC/IF, IP.)
https://www.abcam.cn/products/primary-antibodies/caspase-9-antibody-epr18107-ab202068.html
anti-PARP-1 (Cell Signaling Technology, Cat: 9532, clone: 46D11) (Species reactivity: Human, Mouse, Rat, Monkey. Application: WB, W-S, IP.)
https://www.cellsignal.cn/products/primary-antibodies/parp-46d11-rabbit-mab/9532?site-search-type=Products&N=4294956287&Ntt=9532&fromPage=plp&_requestid=1007293
anti-p-p53 (Cell Signaling Technology, Cat: 9284) (Species reactivity: Human, Mouse, Rat, Monkey. Application: WB, IP, ChIP.)
https://www.cellsignal.cn/products/primary-antibodies/phospho-p53-ser15-antibody/9284?site-search-type=Products&N=4294956287&Ntt=9284&fromPage=plp&_requestid=1007416
anti-F4/80 (Cell Signaling Technology, Cat: 70076, clone: D2S9R) (Species reactivity: Mouse. Application: IP, WB, IHC.)
https://www.cellsignal.cn/products/primary-antibodies/f4-80-d2s9r-xp-rabbit-mab/70076?site-search-type=Products&N=4294956287&Ntt=70076&fromPage=plp&_requestid=1007521
anti-TRAIL (Proteintech, Cat: 27064-1-AP) (Species reactivity: Human. Application: IF, WB.)
https://www.ptgcn.com/products/TRAIL-Antibody-27064-1-AP.htm

Eukaryotic cell lines

Policy information about cell lines and Sex and Gender in Research

Cell line source(s)	Human kidney tubular cell line HK-2 (GDC0152, from China Center for Type Culture Collection, CCTCC, Wuhan, China) was cultured in DMEM/F12 medium (Hyclone, Logan, UT) with 10% fetal bovine serum. Mouse kidney tubular cell line TCMK-1 (CCL-139, from National Collection of Authenticated Cell Cultures, Shanghai, China) and human embryonic kidney cell line HEK293T (CL-0005, from Procell Biotech, Wuhan, China) were cultured in DMEM/High glucose (Hyclone) and 10 % fetal bovine serum.
Authentication	HK-2, TCMK-1 and HEK293T cells were analyzed with authenticated STR locus and tested for mycoplasma contamination, by CCTCC or National Collection of Authenticated Cell Cultures or Procell Biotech.
Mycoplasma contamination	Cell lines routinely monitored for mycoplasma and were all mycoplasma-negative.
Commonly misidentified lines (See ICLAC register)	No commonly misidentified cell lines were used.

Animals and other research organisms

Policy information about studies involving animals; ARRIVE guidelines recommended for reporting animal research, and Sex and Gender in Research

Laboratory animals	Species: mouse<br>Gender: male<br>Age: 8-12 weeks old<br>Mouse strains, including Kim1-flox mice (Gempharmatech, Nanjing, China) and Ksp-Cre mice (CDH16-Cre, Cre activity expressing in renal tubules; from Dr. Congyi Wang, Huazhong University of Science and Technology) were used to generate renal tubule cell-specific Kim1 knockout mice. C57BL/6 mice were purchased from Hubei Provincial Center for Disease Control and Prevention (Wuhan, China).
Wild animals	No wild animals were used.
Reporting on sex	The male mice were used for this study.
Field-collected samples	No field-collected samples were used.
Ethics oversight	All animals were performed in accordance with a protocol approved by the Committee on Ethics in the Care and Use of Laboratory Animals of College of Life Sciences, Wuhan University.

Note that full information on the approval of the study protocol must also be provided in the manuscript.

Flow Cytometry

Plots

Confirm that:

☒ The axis labels state the marker and fluorochrome used (e.g. CD4-FITC).

☒ The axis scales are clearly visible. Include numbers along axes only for bottom left plot of group (a 'group' is an analysis of identical markers).

☒ All plots are contour plots with outliers or pseudocolor plots.

☒ A numerical value for number of cells or percentage (with statistics) is provided.

Methodology

Sample preparation	Cells subjected to different treatments were labeled with FITC-Annexin V and propidium iodide (PI) with an apoptosis detection kit (BB-412, Best Bio., Shanghai, China).
Instrument	BD FACSAria flow cytometer (BD, Franklin Lakes, NJ).
Software	The results were analysed with FlowJo V10 (BD) software.
Cell population abundance	The cell population abundance was determined by cell counting.
Gating strategy	The FITC-Annexin V and propidium iodide (PI) gate were identified basing on compensated monopositive tube control.

☐ Tick this box to confirm that a figure exemplifying the gating strategy is provided in the Supplementary Information.
